# Screening of Mood Symptoms Using MMPI-2-RF Scales: An Application of Machine Learning Techniques

**DOI:** 10.3390/jpm11080812

**Published:** 2021-08-20

**Authors:** Sunhae Kim, Hye-Kyung Lee, Kounseok Lee

**Affiliations:** 1 Department of Psychiatry, Hanyang University Medical Center, Seoul 04763, Korea; sunhk0906@hanyang.ac.kr; 2 Department of Nursing, College of Nursing and Health, Kongju National University, Kognju 32588, Korea; hklee@kongju.ac.kr

**Keywords:** Minnesota Multiphasic Personality Inventory, depression, bipolar disorders, risk factors, machine learning, Patient Health Questionnaire, mood disorder questionnaire

## Abstract

(1) Background: The MMPI-2-RF is the most widely used and most researched test among the tools for assessing psychopathology, and previous studies have established its validity. Mood disorders are the most common mental disorders worldwide; they present difficulties in early detection, go undiagnosed in many cases, and have a poor prognosis. (2) Methods: We analyzed a total of 8645 participants. We used the PHQ-9 to evaluate depressive symptoms and the MDQ to evaluate hypomanic symptoms. We used the 10 MMPI-2 Restructured Form scales and 23 Specific Problems scales for the MMPI-2-RF as predictors. We performed machine learning analysis using the k-nearest neighbor classification, linear discriminant analysis, and random forest classification. (3) Results: Through the machine learning technique, depressive symptoms were predicted with an AUC of 0.634–0.767, and the corresponding value range for hypomanic symptoms was 0.770–0.840. When using RCd to predict depressive symptoms, the AUC was 0.807, but this value was 0.840 when using linear discriminant classification. When predicting hypomanic symptoms with RC9, the AUC was 0.704, but this value was 0.767 when using the linear discriminant method. (4) Conclusions: Using machine learning analysis, we defined that participants’ mood symptoms could be classified and predicted better than when using the Restructured Clinical scales.

## 1. Introduction

The Minnesota Multiphasic Personality Inventory-2 (MMPI-2) was developed to discriminate and diagnose commonly occurring mental disorders. The MMPI-2 is the most widely used tool in personnel screening for occupations that require appropriate psychological coordination and responsibility, such as police officers, firefighters, air traffic controllers, and flight attendants [1,2,3,4,5,6]. The reason for the widespread use of the MMPI-2 in personnel review is its relatively easy management, objective scoring and interpretation procedures, and validity scales of clinically relevant symptomatic behaviors obtained from diverse populations [7].

As such, the MMPI-2 has been the subject of various validity studies and has the advantage of being able to use validity scales to detect response bias not only in psychiatry, but also in forensic evaluation and daily screening tests [8]. Additionally, the use of the MMPI-2 in the differential diagnosis of psychiatric disorders contributes to large-scale research-based evidence of its validity [9].

Mood disorders, including major depressive disorder (MDD) and bipolar disorder, are among the most common mental disorders [10]. They cause social dysfunction due to frequent relapses and represent major causes of suicide [11,12,13].

Many who suffer from mood disorders make repeated primary care visits without receiving an accurate diagnosis. Among low-income individuals, patients with mood disorders are evaluated by an average of eight physicians before receiving a correct diagnosis [14]. Moreover, symptoms began at least 10 years before the average patient was diagnosed with a mood disorder [15]. Because primary care detects only 30–50% of mood disorders, patients with these disorders often remain untreated [16,17,18,19,20], which can lead to detrimental outcomes such as mood instability or deterioration, and even suicide [19,21].

Prevention of mood disorders may be possible through early screening and intervention [22,23,24,25]; therefore, accurate diagnosis of mood disorders is vital in providing appropriate treatment [26,27,28,29]. Many studies have demonstrated that early diagnosis and treatment of mood disorders improves outcomes dramatically [30,31,32], and efficient screening tools allow both mental health care providers and researchers to identify and assist those most likely to benefit from a full psychiatric assessment. This can also help streamline treatment resources.

However, the early detection of mood disorders also entails the necessity of discriminating between depressive and bipolar disorders. Early detection of bipolar patients is difficult because they spend much more time in the depressive phase than in the manic or hypomanic phase [33]. Individuals with bipolar disorder are more likely to seek help for depression than for hypomania or mania because the former’s symptoms tend to be more distressing [20]. This tendency can lead to misdiagnosis of bipolar disorder as depressive disorder. Such misdiagnosis and subsequent inappropriate treatment (e.g., antidepressant-induced manic switch) can lead to detrimental outcomes such as increased suicide risk and mood instability [19,21]. These outcomes mirror symptoms of depressive disorder, as depression often also entails great personal suffering, increased risk of suicide, and social dysfunction [14,34,35,36,37,38,39].

Late diagnosis of these mood disorders can worsen the prognosis [40]. As treatment options for mood disorders are currently available in primary care settings but rarely used in a timely manner due to late diagnosis, new strategies to improve mood disorder detection in primary care settings are urgently needed [41]. Because accurate diagnosis of mood disorders is important for providing adequate treatment and early diagnosis and treatment yield much better outcomes [30,31,32], efficient screening tools, such as full psychiatric assessments, can benefit both mental health care providers and researchers [26,27,28,29]. Efficient screening tools can identify and assist those who need treatment most and streamline treatment resources.

However, despite recognizing the importance of early diagnosis, diagnosis is still unclear. Furthermore, despite significant progress over the last 100 years in clinical psychology and psychiatry [42,43,44], prognosis is still uncertain [45]. In psychiatry, machine learning (ML) research has been conducted for more than 20 years [46], and the advances in algorithms and computing power over the past decade have enabled even more precise research. ML is useful when analyzing multiple predictors, especially when there are nonlinear observations and interaction terms that are difficult to conceptualize theoretically and model in practice. One Danish study used ML to determine predictors of suicide using patient datasets from national medical registries [47]. This study highlights the importance of mental illness as a risk factor for suicide and reveals that ML-based predictive models can improve the accuracy and usefulness of tools developed for specific diagnostic groups.

ML-based predictive models are becoming increasingly popular due to their ability to combine large amounts of data into a single model and their self-evaluation of predictive value for previously unseen patients. Specifically, researchers have used ML methods successfully to predict the persistence, duration, and severity of major depressive disorder [48], as well as treatment responses [49], suicide attempts [50], and first onset of major depressive episodes in US soldiers [51,52]. ML methods are especially useful in fields where evaluation is complex, time-consuming, and expensive, such as when a differential diagnosis is required because the initial diagnosis is unclear [53]. In this study, we explore the MMPI-2′s effectiveness in screening for mood disorders and whether it can be improved through ML techniques with predictive efficiency.

## 2. Materials and Methods

### 2.1. Participants

This study used a dataset from a survey conducted by Kongju National University across South Korea [54]. The researchers collected data from 8882 total participants, excluding from analysis 237 responses from those who did not complete the questionnaire or did not meet the requirements for the MMPI-2 validity scale. Additionally, participants who completed the full MMPI-2-RF scales, PHQ, and MDQ scales were included (response rate = 97.33%). The participants were healthy university students, 18.94 years old (SD = 1.64), and 4274 of them were female. All participants provided written informed consent, and the researchers assured the anonymity of the results and that the data would be used strictly for research purposes. This study was approved by the Research Ethics Committee of Kongju National University.

### 2.2. Psychopathology Assessment Method

#### 2.2.1. MMPI-2-RF (Minnesota Multiphasic Personality Inventory-2-Restructured Form)

The MMPI-2 Restructured Form (MMPI-2-RF) [55,56] emerged after the development of the MMPI-2. It is shorter than the MMPI-2 (338 items instead of 567) and does not include existing clinical and content measures from the MMPI-2. Instead, the Restructured Clinical (RC) scale constitutes the MMPI-2-RF’s main content [57]. The MMPI-2-RF contains one new scale, seven revised Validity scales, three Higher Order scales based on the RC scale, 23 Specific Problem scales, two Interest scales, and five revised Personality Psychology Five (PSY-5) scales. Because the MMPI-2-RF is shorter and demonstrates greater internal consistency than the original clinical scale, it is better at identifying differences and similarities between cultures and has more advantages considering the large number of available translations. The internal consistency of the MMPI-2-RF differs across groups and items; nevertheless, it has good overall internal consistency (Cronbach’s α = 0.76–0.93) [58,59], and many studies have utilized the measure. In this study, we used the measure’s 10 Restructured Clinical (RC) scales and 23 Specific Problems (SP) scales as predictors.

The RC scale was developed by Tellegen (2003) et al. to solve the problems of the clinical scale of MMPI-2-RF and the difficulty in interpretation [57]. Ben-Proath and Tellegen recommend interpreting factors by RC scales [55], and by fixing the higher-order structure in the historical outline of personality structure studies, the analysis of RC scales is further confirmed [60]. Within this framework, interpretive information from each of the RC scales is organized, along with the specific problems (SP) scales associated with each RC scale. The RC scale separates the variable of Demoralization (RCd) that is generally reflected in the clinical scale, and extracts and scales the unique key factors measured in each clinical scale. As the convergent validity and discriminant validity of the RC scale improved compared to the clinical scale, it contributed greatly to resolving the ambiguity in the interpretation of the clinical scale. Additionally, RC scales are a more discriminating and predictive measure that can be used independently as empirical studies are accumulated [61].

The 23 Specific Problems scales are stand-alone scales constructed to either highlight important characteristics contained by or associated with the RC scales or to measure unique constructs not assessed by those mid-level scales [56]. The SP scales are divided into four subsets: Somatic/Cognitive, Internalizing, Externalizing, and Interpersonal Problems scales. The five Somatic/Cognitive Problems scales were derived primarily form Somatic Complaints (RC1). The nine Internalizing Problems scales were designed to measure components of Demoralization (RCd) and Dysfunctional Negative Emotions (RC7). Four scales (i.e., Suicidal/Death Ideation, Helplessness/Hopelessness, Self-Doubt, and Inefficacy) assess facets of RCd, whereas five scales (i.e., Stress/Worry, Anxiety, Anger Proneness, Behavior-Restricting Fears, and Multiple Specific Fears) assess aspects of RC7 [62].

#### 2.2.2. MDQ (Mood Disorder Questionnaire)

The Mood Disorder Questionnaire (MDQ) is a self-report that can be quickly and easily scored by a doctor, nurse, or trained medical assistant. The MDQ screens for a lifetime history of manic or hypomanic syndrome, including 13 yes/no items derived from DSM-IV criteria and clinical experience. The yes/no question also asks whether several of the reported manic or hypomanic symptoms or behaviors were experienced during the same period [63].

The MDQ comprises 13 items (Criterion 1) asking whether one has experienced symptoms of mania or hypomania in one’s lifetime. Next, there are items evaluating how many of these symptoms occurred simultaneously (Criterion 2) and how many functional problems resulted from the symptoms (Criterion 3). All Criterion 1 items utilize a yes/no response format, with the sum of the “yes” answers constituting the total score. For Criterion 3, responses are measured on a 4-point scale ranging from “no problem” to “serious problem”. The original authors state that if the respondent answers “yes” to more than seven of the 13 items and these symptoms appeared at the same time and caused more than moderate problems and dysfunction, then Criteria 1, 2, and 3 were all met. In such cases, there is a strong possibility of bipolar spectrum disorder. In the original development study, the MDQ showed good internal consistency (Cronbach’s α = 0.90, and the item–total correlation coefficients ranged from 0.50–0.75 [63]. The validation study for the Korean version of the measure also indicated good internal consistency (Cronbach’s α = 0.88) [64].

#### 2.2.3. PHQ-9 (The Patient Health Questionnaire)

The PHQ-9 is a part of the PHQ (Patient Health Questionnaire), which is a self-reporting questionnaire developed in order to detect and diagnose mental illnesses in a primary clinical setting. The PHQ-9 is designed to be consistent with DSM-IV depression diagnostic criteria for the selection of major purposes; there is a total of nine questions, and the end of the daily life due to symptoms is added. We have considered that 9 points were used as a cut-off point in the evaluation using the total score of the PHQ-9 [65].

### 2.3. Procedure

We used 33 total scales (10 RC scales and SP scales) as predictors. To compare the discriminating ability with the existing clinical scale, we analyzed the receiver operating characteristic (ROC) curve with the demoralization RC scale (RCd) for depressive symptoms and the hypomanic activation T-score (RC9) of 65 for hypomanic symptoms as the cutoff points. The ROC curve is a plot that displays the full picture of trade-off between sensitivity and (1-Specificity) over a series of cut-off points. The area under the receiver operating characteristic curve (AUC) is considered as an effective measure of inherent validity of a diagnostic test [66]. Therefore, in this study, a diagnostic analysis was performed with the AUC value using the ROC curve. For ML methods, we used the k-nearest neighbor classification (KNN), linear discriminant analysis (LDA), and random forest classification. All data analysis took place within JASP v0.14.4 (Amsterdam, the Netherlands) and MedCalc v20.009 (MedCalc Software, Mariakerke, Belgium).

### 2.4. Computational Analyses

Random forest is an ensemble learning method composed of multiple decision trees [67]. Decision trees allow simple and fast learning and testing by simply dividing complex problems in a hierarchical structure, and they show high accuracy and generalization performance by constructing an arbitrary decision tree and performing ensemble learning. The ensemble learning method allows for learning new hypotheses by creating multiple classifiers and combining their predictions. The goal of ensemble learning is to obtain high reliability prediction values compared to a single classifier by synthesizing the prediction results of multiple classifiers. Random forest performs ensemble learning using bootstrap aggregating (bagging). Bagging constructs many sample sets by applying a bootstrapping sampling method that allows duplicates to the dataset to be used for analysis, using each sample set as a training dataset to create a classifier set and then creating a complex classifier.

The KNN is one nearest-neighbor search technique. It is an optimization method of unsupervised learning that finds a solution in given k-nearest neighbors. As a method of classifying new data values as a criterion, a majority vote determines the group. As an algorithm with a relatively simple structure, estimates use only local information, and “lazy learning” that can be classified after the calculation of the entire space is completed by the lazy learning method. This method has the advantage of being efficient and simple to implement, even when the training dataset is large or contains much noise. Additionally, then, the KNN algorithm has been presented as a good machine learning method in previous studies to discriminate mood disorders [68,69,70].

As a dimension-reduction algorithm, LDA finds a vector that maximizes the difference between the average values of each class and minimizes the variance [71]. Therefore, LDA aims to achieve maximum class discrimination by finding optimal discriminant vectors (transformation) in maximizing the ratio of between-class and within-class distances [72]. As LDA considers the correlations between categorical variables and already correlated variables [73], we selected the minimum set of weakly correlated and potentially discriminable variables according to the results. The study sample was divided into two subsets and used to test the model’s ability to discriminate among depressive, hypomanic, and symptom-free groups.

## 3. Results

### 3.1. Experimental Results

#### 3.1.1. General Characteristics

Of the 8645 total responses, we used 5532 as training data, 1384 as validation data, and 1729 as test data. The average age of the sample was 18.92 years (±1.64). Women accounted for 4273 responses (36.9%). A total of 1116 participants (9.6%) exhibited depressive symptoms, and 1068 (9.2%) exhibited hypomanic symptoms (Table 1).

#### 3.1.2. Diagnostics Characteristics of MMPI-2-RC Scales

When using RCd to predict depressive symptoms, the sensitivity was 90.43% (95% CI = 89.76–91.07), PPV was 95.64 (95% CI = 95.06–96.09), and the area under the curve (AUC) was 0.807; however, when using LDA, the AUC was 0.840. When predicting hypomanic symptoms using RC9, the sensitivity was 88.83% (95% CI = 88.13–89.5), PPV was 96.23 (95% CI = 95.77–96.64) and the AUC was 0.704; in contrast, when using KNN, the AUC was 0.767 (Table 2).

#### 3.1.3. Mood Disorders Item Combination by ML Technique

For both depressive and hypomanic symptoms, and for each of the three ML techniques, the accuracy was more than 87% (87.3–88.5%). Each ML algorithm showed high sensitivity (88.13–91.71%) and PPV (95.58–99.60%), with LDA showing a sensitivity of 91.71% (95% CI = 90.24–93.02) for depression and KNN showing sensitivity for hypomanic symptoms. LDA resulted in the best sensitivity of 89.29% (95% CI = 87.72–90.72; Table 3).

## 4. Discussion

In this study, we evaluated the MMPI-2′s effectiveness in screening for mood disorder symptoms. The instrument yielded high accuracy for both depressive and hypomanic symptoms (87.3–88.5%) when using ML techniques, and the AUC was 0.634–0.840, indicating excellent predictive diagnostics.

The MMPI-2-RF consists of 50 scales, including 10 RC scales that address the limitations of the MMPI-2 clinical scale, and has 229 fewer items than the MMPI-2. The RC scale has significantly improved discriminative validity compared to the original clinical scale. Among them, researchers have studied RCd and RC9 to measure mood symptoms more specifically [74,75]. Typically, the RCd scale measures depressive symptoms and the RC9 scale measures hypomanic symptoms. The RCd and RC9 scales have predicted depressive and hypomanic symptoms of mood disorders with a high score for AUC and Sensitivity, respectively (RCd = 81% AUC, 90.43 sensitivity; RC9 = 70% AUC, 88.83 sensitivity).

In this study, we used ML techniques to examine the relationship between these RC scales and mood symptoms. Compared with the RCd of the MMPI-2-RF (AUC = 0.81), ML-based analysis yielded more accurate predictive values, with the exception of KNN (KNN = 77%, LDA = 74%, RF = 83%). In the case of hypomanic symptoms, when analyzing the RC9 items (AUC = 0.70), the ML techniques showed superior results compared to the traditional statistical techniques, except again for KNN (KNN = 63%, LDA = 77%, RF = 73%). This means that ML-based analysis can facilitate more sophisticated clinical interpretation of the MMPI-2 than analysis using existing clinical scales or content scales and scale pairs. ML algorithms are designed to maximize clinical significance and generalizability, and are well equipped to process a large number of variables as potential predictors [76]. Herein, using ML techniques, we evaluated the predictive value of screening which was analyzed through RCd and PHQ in the MMPI-2-RF scale for depressive symptoms among mood disorders and through RC9 and MDQ for hypomanic symptoms.

The introduction of ML into statistical analysis in psychiatric research represents a paradigm shift beyond simply adding analytical tools for combining and exploring larger datasets [77]. For many years, classical statistical approaches have been used to confirm or refute certain hypotheses, but modern ML research focuses on the overall predictive power of models, especially on how accurately they predict desired outcomes in new and unencountered datasets. Research in this field is primarily evaluated by its potential clinical impact and whether it can provide reliable information about the prognosis for future patients (especially when patients go unscreened and undiagnosed for many years), leading to treatments and interventions [49]. It will be a useful predictive technique for detection-resistant mood disorders.

The advantage of using the MMPI-2-RF as a screening tool for mood disorders as performed in this study is that it may be a more efficient method of collecting various clinical data simultaneously [9]. Additionally, the MMPI-2-RF takes less than 30 min to complete on a computer, which is more efficient than unstructured interviews and some other self-report tools. This is true even considering that the most widely used screening tool for bipolar disorder, the MDQ, cannot adequately differentiate between bipolar disorder and borderline personality disorder [78]. Unlike most other measurement tools, the MMPI-2-RF can increase diagnostic reliability by including an effectiveness scale to detect underreporting and overreporting of psychiatric symptoms.

The MMPI-2 may also be useful for differential diagnosis of clinical patients with mood disorders. Five of the MMPI-2-RF scales showed significant differences between patients diagnosed with BD and those with MDD. The Activation (ACT) scale had the largest AUC (0.74) and effectively discriminated BD and MDD patients. Overall, 71% of patients were correctly classified according to the ACT scale, and 72% of patients currently experiencing depression were correctly classified. This is superior to the 66% classification rate found in a previous study of SAD-P using a tool other than the MMPI-2-RF [79]; further, because bipolar disorder uses a major depressive episode as a common diagnostic criterion, it can be compared with major depressive disorder. Because discrimination of mood disorders is important, the discriminating ability of the MMPI-2-RF scale suggests its strength as a measurement tool. This study examined the MMPI-2-RF’s application as a screening tool, demonstrating that the existing RCd and RC9 also account for considerable AUC (0.807 and 0.704, respectively).

This study has several limitations. First, a study using the results of student health examinations lacks clinical diagnoses. The gold standard is analyzing diagnoses using structured interviews or clinical evaluations, but we used the results of the PHQ-9 and MDQ. The symptom evaluation was performed by scale; even if the scores are the same, it does not mean that they have the same symptoms. In future studies, it may be helpful to subclassify the symptom groups and analyze the symptom features. However, as a tool for screening mood disorder symptoms, our study still suggests the appropriateness of this measure’s application. Second, there was no information on participants’ clinical histories. Third, our sample of students from a single university may not represent the entire population. However, by securing a sufficiently large number of participants, we argue that there is a certain degree of generalizability with respect to the college student population. Fourth, while the machine learning techniques used in this study have high AUC and sensitivity, they show low specificity, so in future research, theoretical implications or mood disorder tools with high all statistical values should be verified. In addition, future studies use the statistical strength of this paper to recommend empirical studies that include two subgroups for screening for mood disorders (e.g., healthy individuals versus individuals with clinical diagnosis of an affective disorder). Additionally, through other ML techniques (e.g., unsupervised ML techniques, reinforcement learning), future studies could present which features were important based on the machine learning techniques in mood disorders.

In this study, we determined the reliability of the test using the RC scale of the MMPI-2-RF. The advantage of the self-report test is that it may be easier to communicate emotional difficulties compared to in interviews, but it is difficult to ensure reliability. This study was able to reasonably ensure reliability by using the clinically reliable MMPI-2-RF assessment. The MMPI-2 is widely used at selection and interview sites, and analysis using ML techniques suggests the appropriateness of using it for screening high-risk groups as well as for existing clinical and content measures. Additionally, because it is possible to identify emotional states without directly asking about emotional difficulties, it will be helpful in screening clinical symptoms in non-clinical sites (e.g., screening tests). In addition to the existing MMPI interpretation methods used in the clinical field, prediction-based ML techniques can help screen for mood disorders.

## 5. Conclusions

We have confirmed that ML techniques exhibit excellent predictive power compared with the RC scales of the MMPI-2-RF. If such techniques are applied to the interpretation of the MMPI-2-RF, it will be possible to detect various psychopathologies early, which is crucial for effective treatment.

## Figures and Tables

**Table 1:** Basic characteristics of participants (*n* = 8645).

Variables		Values
age		18.92 ± 1.64
gender	male	4371 (73.7%)
	female	4274 (36.9%)
PHQ (+)		1116 (9.6%)
MDQ (+)		1068 (9.2%)

PHQ (+): Patient Health Questionnaire screening positive; MDQ (+): Mood Disorder Questionnaire screening positive.

**Table 2:** Diagnostic Characteristics of Reconstructive Clinical Scales.

	AUC	Sensitivity (%)	Specificity (%)	PPV (%)	NPV (%)
RC9	0.704	88.83 (88.13–89.5)	34.55 (30.1–39.22)	96.23 (95.77–96.64)	14.14 (12.1–16.37)
RCd	0.807	90.43 (89.76–91.07)	51.91 (48.08–55.71)	95.64 (95.16–96.09)	31.72 (29.0–34.54)

AUC: Area Under the ROC Curve; PPV: Positive Predictive Value; NPV: Negative Predictive value.

**Table 3:** Measurement characteristics according to machine learning techniques.

	ML Methods	Accuracy	F1 Score	AUC	Sensitivity (%)	Specificity (%)	PPV (%)	NPV (%)
MDQ	KNN	0.880	0.843	0.634	89.29 (87.72–90.72)	33.33 (19.09–50.22)	98.31 (97.53–98.89)	6.70 (3.62–11.19)
	Linear Discriminant	0.873	0.743	0.767	89.06 (87.46–90.52)	43.94 (31.74–56.7)	97.56 (96.66–98.28)	13.74 (9.4–19.14)
	Random Forest	0.879	0.832	0.727	88.13 (86.5–89.62)	68.42 (43.45–87.42)	99.60 (99.14–99.85)	6.02 (3.24–10.07)
PHQ	KNN	0.883	0.848	0.770	88.66 (87.05–90.13)	75.56 (60.46–87.12)	99.27 (98.7–99.63)	15.11 (10.7–20.47)
	Linear Discriminant	0.885	0.877	0.840	91.71 (90.24–93.02)	55.03 (46.68–63.18)	95.58 (94.42–96.56)	38.5 (31.93–45.39)
	Random Forest	0.879	0.855	0.828	89.67 (88.09–91.1)	52.38 (41.19–63.4)	97.36 (96.42–98.11)	20.56 (15.35–26.6)

## Data Availability

The data presented in this study are available on request from the corresponding author. The data are not publicly available due to ethical restrictions.
